# Type I Interferons as Stimulators of DC-Mediated Cross-Priming: Impact on Anti-Tumor Response

**DOI:** 10.3389/fimmu.2013.00483

**Published:** 2013-12-25

**Authors:** Giovanna Schiavoni, Fabrizio Mattei, Lucia Gabriele

**Affiliations:** ^1^Department of Hematology, Oncology and Molecular Medicine, Istituto Superiore di Sanità, Rome, Italy

**Keywords:** type I interferon, interferon alpha, cross-priming, dendritic cells, dendritic cell subsets, cancer, tumor-associated antigen, immunosurveillance

## Abstract

Induction of potent tumor-specific cytotoxic T-cell responses is a fundamental objective in anticancer therapeutic strategies. This event requires that antigen-presenting cells present tumor-associated antigens (Ag) on their MHC class-I molecule, in a process termed cross-presentation. Dendritic cells (DC) are particularly keen on this task and can induce the cross-priming of CD8^+^ T cells, when exposed to danger or inflammatory signals that stimulate their activation. Type I interferons (IFN-I), a family of long-known immunostimulatory cytokines, have been proven to produce optimal activation signal for DC-induced cross-priming. Recent *in vitro* and *in vivo* evidences have suggested that IFN-I-stimulated cross-priming by DC against tumor-associated Ag is a key mechanism for cancer immunosurveillance and may be usefully exploited to boost anti-tumor CD8^+^ T-cell responses. Here, we will review the cross-presentation properties of different DC subsets, with special focus on cell-associated and tumor Ag, and discuss how IFN-I can modify this function, with the aim of identifying more specific and effective strategies for improving anticancer responses.

## Introduction

Anti-tumor immune responses are evoked by several effector cells. These include both innate immune cells, like NK cells and macrophages, and cells of the adaptive immunity. Among these, CD8^+^ T cells are ideal tumoricidals, due to their capacity to recognize and kill malignant cells in an antigen (Ag)-specific fashion and to establish a long-lasting protection. The activation of anti-tumor CD8^+^ T-cell responses is fulfilled through a process known as cross-priming and requires the uptake of extracellular Ag also in the form of tumor cells by the antigen-presenting cell (APC), which subsequently delivers the engulfed material to a distinct endosomal/lysosomal pathway that allows the processed peptides to be presented on MHC class-I (MHC-I) molecules (cross-presentation) (1).

Among APC, dendritic cells (DC) have been described as the sole cell type able to cross-present cell-associated Ag and studies on both mouse and human models have revealed that distinct DC subsets display differential capacities to perform this process resulting in the induction of immunity or tolerance. In this respect, for cross-presentation to result in cross-priming, three signals must be delivered by DC: (i) loading and cross-presentation of cell-derived Ag onto MHC-I, (ii) appropriate co-stimulation through membrane molecules, and (iii) secretion of pro-inflammatory cytokines. Among cytokines produced by DC and capable of triggering DC activation, type I interferons (IFN-I) have been shown to play a major role in promoting cross-priming against both soluble proteins and cell-associated Ag, such as Ag derived from tumor apoptotic cells.

Here we discuss the most recent advances in Ag cross-presentation properties by several types of DC and on the capacity of IFN-I to turn on CD8^+^ T-cell cross-priming.

## DC Subsets Capable of Mediating Cross-Priming

### Mouse DC

In the murine immune system several DC subtypes have been characterized (2). The spleen contains at least five subsets distinguished by expression of specific surface markers: plasmacytoid DC (pDC; CD11c^low^PDCA-1^+^B220), CD8α DC (CD8α^+^CD4^−^CD11b^−^), CD11b DC (CD8α^−^CD4^−^CD11b^+^), CD4 DC (CD8α^−^CD4^+^CD11b^+^), and merocytic DC (mcDC; CD8α^−^CD4^−^CD11b^−^). These DC subsets markedly differ in their abilities to capture and cross-present antigenic material and only some of them can cross-present cell-associated Ag (3).

CD8α DC is the most efficient DC subset in Ag cross-presentation uniquely able to prime CD8^+^ T cells against cell-associated Ag *in vivo* (4–6). In the steady-state, CD8α DC capture dead cells resulting from constitutive turnover and play a central role in self-tolerance (6, 7). The *in vivo* relevance of CD8α DC in CD8^+^ T-cell cross-priming against cell-associated Ag has been better clarified by studies with mice devoid of this DC subset. Mice deficient for either transcription factors Batf3 or NFIL3/E4BP4, both lacking CD8α DC selectively, display impaired cross-priming of CD8^+^ T cells against cell-associated Ag (8, 9). Similarly, IRF-8^−/−^ mice, which are devoid of CD8α DC and pDC, display impaired capacity to cross-present both soluble and tumor cell-derived Ag (10, 11).

Initial studies showing that CD8α DC capture cellular Ag more efficiently than other DC subsets suggested that this was the principal mechanism for increased cross-presentation ability by CD8α DC (5, 12). Indeed, CD8α DC selectively express some receptors, such as CLEC9A or Tim-3, involved in the recognition of necrotic and apoptotic cells, respectively, and implicated in cross-presentation of cellular Ag (13–15). Additional studies unraveled that CD8α DC also possess a special processing machinery that delivers the internalized Ag onto the MHC-I processing pathway (16). Such machinery involves the activity of the small GTPase Rac2, selectively operating in CD8α DC, and the subcellular assembly of the NADPH oxidase complex (NOX2) to phagosomes that maintains a high phagosomal pH and thus facilitates cross-presentation (17).

The lymph nodes (LN) contain additional DC subtypes, termed migratory DC, arising from non-lymphoid tissues where they normally reside. Of the two types of migratory DC described, namely CD103^−^CD11b^+^ and CD103^+^CD11b^−^, only the latter has been described to cross-present cellular Ag captured either in the lung (18) or in the skin (19). The shared efficiency for Ag cross-presentation by CD8α DC and CD103^+^ DC has been attributed to a developmental relationship, since these two DC subsets have a common dependence on the transcription factors Batf3, Flt3L, Id2, and IRF8 for their differentiation (20–22). Recent findings showed that CD8α DC and CD103^+^ DC specifically co-express XCR1, a receptor for CD8^+^ T-cell-secreted XCL1 that couples DC cross-presentation to induction of CD8^+^ T-cell immunity (23, 24). XCR1 was found to be a conserved specific marker also for additional murine DC subtypes (including a small percentage of mcDC and of CD103^−^ DC) and for human DC subsets devoted to cross-presentation of cell-associated Ag (25–27).

Janssen’s group reported that mcDC capture dying cells, although less efficiently than CD8α DC, and cross-prime CD8^+^ T cells for an extended time due to prolonged Ag storage (3, 28). *In vivo*, mcDC induce tumor-specific CTL responses in B16 melanoma-bearing mice (28). Of note, injection of tumor vaccine-loaded mcDC, but not of CD8α DC, elicited protective responses from subsequent tumor challenge in mice in a vaccination EL-4 thymoma model and resulted in therapeutic eradication of established EL-4 and B16 melanoma tumors (28, 29).

Although cross-presentation of soluble proteins by mouse pDC can occur upon Toll-like receptor (TLR) engagement (30), there is no evidence that pDC may cross-present cell-associated Ag. Instead, pDC can indirectly enhance CD8^+^ T-cell cross-priming, through production of IFN-I and other soluble mediators (31–34). The capacity of CD11b DC to cross-present cellular Ag is also weak. In a murine model of mesothelioma expressing influenza virus hemagglutinin, as a membrane-bound neo-tumor Ag, one group has reported that both CD8α DC and CD11b DC from tumor-draining LN could cross-present membrane hemagglutinin (35). This observation suggests that the anatomical location may affect the efficacy of CD11b DC for tumor Ag cross-presentation.

### Human DC

Human DC also display some heterogeneity. In the blood, DC may be essentially distinguished into BDCA1^+^ myeloid DC (mDC), BDCA3^+^ mDC, and pDC. BDCA3^+^ mDCs have been reported to cross-present Ag on their MHC-I molecules more efficiently than other DC populations. Due to functional and phylogenetic similarities, this subset is thought to be the human equivalent of mouse CD8α DC (36–38). BDCA3^+^ mDCs selectively express CLEC9A and XCR1 and efficiently cross-present Ag derived from dead cells (25, 36, 37).

The role of human pDC as professional APC in the cross-presentation of exogenous Ag is under intensive investigation. Tumor cells infected with a measles virus vaccine are able to induce tumor Ag cross-presentation by human pDC via production of large amounts of IFN-α (39). Furthermore, harnessing uptake receptors to deliver Ag to pDCs can enhance cross-presentation and IFN-I production, resulting in the generation of potent anti-tumor responses (40). The efficacy of pDC has been verified in a cohort of metastatic melanoma patients in whom activated pDC were found to induce Ag-specific T-cell responses and significantly extended overall survival (41). It has been recently shown that pDC cross-present soluble and cell-associated tumor Ag to cytotoxic T lymphocytes to the same degree as BDCA3^+^ mDC (42, 43). Indeed, two recent reports argued on the notion that cross-presentation is restricted to certain human DC subsets. Amigorena’s laboratory showed that freshly isolated tonsil-resident pDC, BDCA1^+^, and BDCA3^+^ mDC cross-present soluble Ag with the same efficiency, displaying comparable phagosomal pH, production of reactive oxygen species and capacity to export internalized proteins to the cytosol (44). Delamarre’s group reported that the diverse human DC subsets are equally able to cross-present exogenous Ag to CD8^+^ T cells provided that the Ag is delivered to early endocytic compartments (43). These findings have extensive implications for vaccination strategies aiming at exploiting *ex vivo*-differentiated autologous DC, resembling primary DC subsets and endowed with strong cross-priming ability.

## Enhancement of Cross-Presentation by IFN-I in Murine DC

In the steady-state or in the context of a tumor, DC cross-presentation of cell-associated Ag rarely results in CD8^+^ T-cell cross-priming due to lack of immunostimulatory signals capable of activating DC. IFN-I is the prototype inflammatory cytokine released upon infection or under physiological distress acting as a stimulus for DC cross-priming (45). *In vivo*, IFN-I induces CD8^+^ T-cell cross-priming against viral or soluble protein Ag through DC stimulation (46). Recently, we showed that IFN-I can affect DC cross-presentation of cell-associated Ag. *In vitro* or *in vivo* exposure of CD8α DC that have engulfed irradiated tumor cells to IFN-I resulted in three distinct effects: (i) increased retention of engulfed apoptotic material that correlated with decreased endosomal acidification and resulted in enhanced Ag cross-presentation, (ii) prolonged survival of phagocytic CD8α DC, and (iii) phenotypic activation of the cross-presenting DC that resulted in DC “licensing” for cross-priming (10). Similar results were obtained using tumor cells killed by the chemotherapeutic agent cyclophosphamide as a source of antigenic material for CD8α DC. In this setting, addition of IFN-I resulted in CD8^+^ T-cell cross-priming *in vitro* and tumor rejection *in vivo* (47).

Two different groups have recently reported the *in vivo* relevance of endogenous IFN-I signaling on CD8α DC for promoting CD8^+^ T-cell-dependent spontaneous tumor rejection. Diamond et al. (48) showed that mice lacking IFN-α/β receptor 1 selectively in DC cannot reject methylcholanthrene-induced fibrosarcoma, a highly immunogenic tumor normally rejected by immunocompetent mice, and that CD8α DC from these mice display defective Ag cross-presentation to CD8^+^ T cells. Similarly, by using IFN-α/β receptor 1^−/−^ and Batf3^−/−^ mice transplanted with B16 melanoma, Fuertes et al. (49) reported that endogenous IFN-I, produced shortly after tumor challenge, was essential for intratumoral accumulation of CD8α DC and for induction of tumor Ag-specific T-cell priming and tumor rejection via CD8α DC stimulation. These studies underscore CD8α DC as fundamental targets for endogenous IFN-I-mediated spontaneous immune control of a rising tumor.

Cross-priming mediated by mcDC also requires IFN-I. However, unlike CD8α DC, which fail to produce IFN-I upon uptake of apoptotic cells, mcDC are able to do so. Adoptive transfer experiments revealed that this endogenous IFN-I acts in an autocrine manner to activate mcDC and is both necessary and sufficient for boosting CD8^+^ T-cell cross-priming against cell-associated Ag (28, 29). Of interest, endogenous IFN-I signaling in mcDC was essential for preserving internalized material from early degradation and endosomal acidification similarly to what observed with CD8α DC exposed to exogenous IFN-I (3, 10). These findings suggest that IFN-I promote cross-priming in DC by exploiting a mechanism involving regulation of endosomal pH and Ag retention that direct the antigenic cargo toward the MHC-I processing pathway, as also observed with human DC (see below) (50). Thus, regulation of phagosomal acidification may be viewed as a strategy exploited by inflammatory signals, such as IFN-I, to switch on cross-priming in those DC subsets that under steady-state are devoted to tolerance induction and may provide a mechanism (coupled to MHC-I up-regulation) by which IFN-I induce autoimmune reactions, namely by enhancing presentation of self Ag.

The ability of some compounds targeting TLR to stimulate CD8^+^ T-cell cross-priming has also been shown to occur through endogenous IFN-I production and subsequent DC stimulation (51, 52). The efficacy of CpG in cancer immunotherapy is dependent on cross-talk between pDC and conventional DC (mcDC and CD8α DC), the first serving as a source of IFN-I through TLR9 triggering and the latter responding to IFN-I to promote CD8^+^ T-cell cross-priming and anti-tumor response in melanoma-bearing mice exposed to cryoablation (53).

## Enhancement of Cross-Presentation by IFN-I in Human DC

Type I interferons exert multiple effects on human DC, affecting the major cellular pathways associated to their APC function, namely differentiation, maturation, and migration (54, 55). Human immature conventional DC treated *in vitro* with IFN-I up-regulate the expression of MHC-I, CD40, CD80, CD86, and CD83 molecules resulting in a superior capacity to induce CD8^+^ T-cell responses (56, 57). Moreover, IFN-I support the differentiation of human monocytes into DC with high capacity for Ag presentation (58). IFNα induces one-step differentiation of human monocytes into highly activated and partially mature DC (IFNα-DC), retaining a marked phagocytic activity and exhibiting a special aptitude for inducing CD8^+^ T-cell responses (59, 60). Studies on phenotype and functions of IFNα-DC have pointed that these cells can resemble naturally occurring DC, generated from monocytes in response to danger signals, including infections when high levels of IFN-I are released (61–65). Indeed, subtypes of DC resembling IFNα-DC have been observed in patients suffering from autoimmune or infectious diseases (54).

IFNα-DC express markers involved in antigen processing such as CD208 and the scavenger receptor oxidized low-density lipoprotein receptor 1 (LOX-1), implicated in Ag uptake and CD8^+^ T-cell cross-priming (66). *In vivo*, IFNα-DC generate cytotoxic responses and CD8^+^ T-cell cross-priming against viral and tumor-associated Ag (59, 67–69). Efficient cross-presentation of tumor-associated Ag by IFNα-DC loaded with apoptotic human melanoma cells was found to correlate with enhanced proteasome activity (68). In addition, studies employing soluble Ag point to an effect of IFNα in preserving Ag from early degradation, thus facilitating its routing onto MHC-I pathway (50). Thus, although the intracellular mechanisms underlying the superior efficiency of IFNα-DC in Ag cross-presentation need to be clarified, these evidences suggest that IFN-I may control this process at diverse levels.

Interestingly, IFNα-DC have been reported to exhibit some phenotypic features of pDC (70). We recently reported that IFNα-DC and pDC share a similar miRNA signature as well as some phenotypic and molecular markers potentially accounting for common functional activities, such as IFN-I production upon viral infection. Moreover, IFN-I was also able to affect some functions of pDC, including the expression of the pDC-associated markers IRF-8 and TLR-9 (71).

## Importance of DC Cross-Priming for Anticancer Immune Responses and Perspectives for Exploitation of IFN-I Potentiating Effect

Several lines of evidence indicate that DC-mediated cross-priming is crucial for anti-tumor immunity (72). First, tumor-infiltrating DC purified from tumor samples have the capacity to cross-present tumor Ag *in vitro* (73). Second, priming *in vivo* of anti-tumor T-cell responses can be abrogated in models in which DC subsets specialized for cross-presentation can be specifically depleted. Indeed, *Batf3*^−/−^ mice are unable to reject highly immunogenic tumors due to defective cross-presentation by *Batf3*^−/−^ DC, reduced tumor-infiltration of CD8^+^ T cells and failure to develop tumor-specific CTL (8).

The therapeutic anti-tumor potential of IFN-I has been appreciated since 1960s (74, 75). However, only recently it has become clearer how IFN-I participate in naturally occurring, protective immune responses to primary tumors, thus playing a prominent role in cancer immunosurveillance. In addition, IFN-I has been shown to be a crucial component of cancer-immunoediting, namely the process whereby the immune system suppresses cancer growth and shapes tumor immunogenicity (76, 77). These findings have renewed the interest in exploiting the anti-tumor potential of IFN-I in therapeutic and vaccination strategies against cancer.

Therapeutic approaches that involve either exogenous IFN-I administration or its induction within the tumor microenvironment have shown effects on CD8^+^ T-cell responses via DC stimulation at various levels. In mice with established B16 tumors, radiotherapy induced a local increase in IFN-I expression by myeloid immune infiltrates that acted enhancing the cross-priming ability of tumor-infiltrating DC and was crucial for host therapeutic response (78). Furthermore, intratumoral delivery of IFN-I synergized with immunotherapy (79) and chemotherapy (47) to induce therapeutic response in tumor-bearing mice that involved, in both cases, enhanced DC cross-presentation. Notably, IFN-I can enhance anti-tumor CTL responses also via direct effects on CD8 T cells, inducing their expansion and acquisition of effector functions thus improving therapeutic efficacy (80, 81).

With regard to protocols employing vaccine preparations, co-administration of CpG with a DC vaccine was found to overcome tumor-specific tolerance after stem cell transplantation, inducing protective anti-tumor response through CpG-induced IFN-I *in vivo* (82). Recently, Shimizu and colleagues showed that vaccination with B16 melanoma cells loaded with the invariant NKT cell ligand αGalCer stimulated tumor-reactive CD8^+^ memory T cells in a novel mechanism involving cross-talk between XCR1-expressing DC and pDC via NKT-stimulated IFN-α production by pDC (33). Human studies also point to the use of IFN-I-inducers as promising approach to boost anti-tumor effector responses. The efficacy of topical application of the TLR7/8 agonist imiquimod, the only TLR agonist approved by FDA for skin cancer treatment, has been linked to local increase of IFN-I production, recruitment of DC and induction of tumor-reactive CTL (83). Finally, it is worth mentioning that tumor-derived IFN-I may also positively contribute to anti-tumor immune response. In virtue of their TLR expression, B16 melanoma cells were found to respond to ligands to TLR3 and TLR4 by releasing substantial levels of IFN-I that induced DC activation and resulted in tumor growth inhibition by the host (84, 85).

## Concluding Remarks

**Figure 1:**
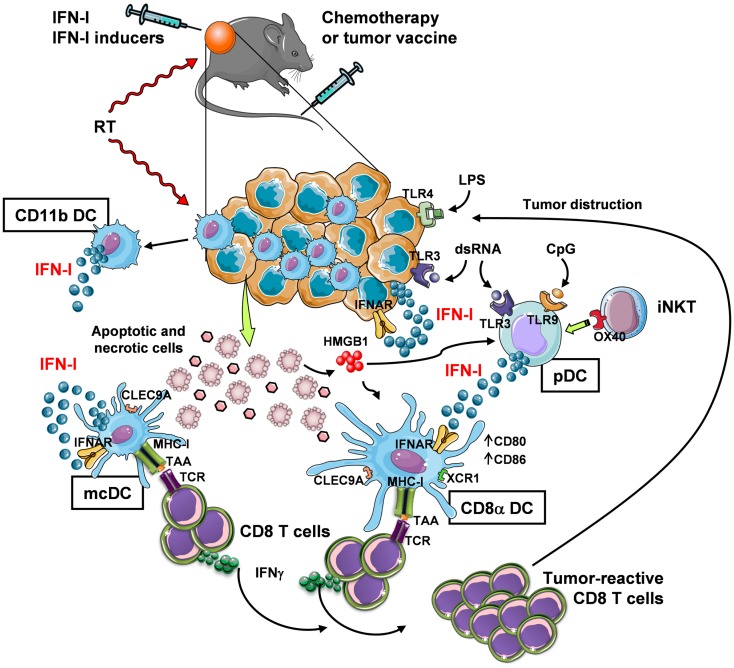
***In vivo* induction of anti-tumor CD8^+^ T-cell responses through IFN-I-mediated DC cross-priming at the tumor site**. Systemic chemotherapy and local radiotherapy (RT) induce tumor cell death that result in the availability of antigenic material (which is otherwise provided by a tumor vaccine) for internalization by specialized DC subsets, namely mcDC and CD8α DC. These subsets then cross-present the tumor-associated Ag (TAA) through their MHC-I complex to CD8^+^ T cells. In order to induce CD8^+^ T-cell cross-priming, the cross-presenting DC need to be exposed to activation stimuli (DC licensing), such as IFN-I. While mcDC spontaneously produce IFN-I that act in an autocrine fashion to induce DC licensing for cross-priming, CD8α DC are unable to do so and require the exogenous cytokine. Thus, in the tumor site IFN-I may be made available in different manners: (1) via intratumoral injection, (2) by RT, which stimulates IFN-I release by infiltrating myeloid CD11b DC (and possibly other immune and non-immune cells), and (3) by intratumoral delivery of IFN-I-inducing substances, such as TLR ligands. Some TLR ligands can also bind to tumor cells that express TLR3 and TLR4 to trigger autocrine IFN-I production and stimulation of DC. Alternatively, TLR ligands, such as dsRNA and CpG, stimulate pDC to produce large amounts of IFN-I. IFN-I secretion by pDC may also be stimulated by invariant NKT (iNKT) cells via OX40 and HMGB1 released by dying tumor cells. The final outcome of these events is the expansion of tumor-reactive CD8 T cells with killing activity.

Despite IFN-α has received approval for therapy of several neoplastic diseases, side effects of systemic long-term treatments and insufficiently high efficacy have challenged its use in current clinical protocols. Therefore, novel strategies to exploit IFN-I in therapeutic and vaccination protocols are needed that take into account, for example, controlled timing of administration to avoid negative feedback mechanisms in the responding immune cells (58, 86), and the involvement of active cross-talk between multiple types of immune cells that play different, non-overlapping roles within the tumor site. In this view, the combined use of chemotherapy or radiotherapy that kill cancer cells, providing source of Ag for DC, with exogenous IFN-I or compounds capable of inducing IFN-I *in situ* may be viewed as promising strategies for boosting DC cross-presentation and CTL induction within the tumor microenvironment (Figure 1).

## Conflict of Interest Statement

The authors declare that the research was conducted in the absence of any commercial or financial relationships that could be construed as a potential conflict of interest.
